# Effect of Filler Type on the Thermo-Mechanical Properties of Metakaolinite-Based Geopolymer Composites

**DOI:** 10.3390/ma13102395

**Published:** 2020-05-22

**Authors:** Jan Kohout, Petr Koutník

**Affiliations:** Unipetrol Centre for Research and Education, Revoluční 84, 400 01 Ústí nad Labem, Czech Republic; petr.koutnik@unicre.cz

**Keywords:** geopolymer, metakaolinite, claystone, thermal properties, mechanical properties

## Abstract

Metakaolinite-based geopolymer binder was prepared at room temperature by mixing calcined claystone and potassium alkaline activator. Various granular inorganic fillers were added, amounting to 65 vol % to form geopolymer composites. The effect of four types of fillers (sand quartz, chamotte, cordierite, and corundum) on the thermo-mechanical properties of metakaolinite-based geopolymer composites were investigated. The samples were also examined by an X-ray diffraction method to determine their phase composition. The pore size distributions were determined by a mercury intrusion porosimeter. The XRD revealed the crystallization of new phase (leucite) after thermal exposure at 1000 °C and higher. Geopolymer binders had low mechanical properties (flexural strength 2.5 MPa and compressive strength 45 MPa) and poor thermo-mechanical properties (especially high shrinkage—total shrinkage 9%) compared to geopolymer composites (flexural strength up to 13.8 MPa, compressive strength up to 95 MPa and total shrinkage up to 1%). The addition of fillers reduced the shrinkage of geopolymers and improved their mechanical properties. The results have shown that the compressive strength tested in situ and after exposure to high temperature are in conflict. Geopolymer composites with the addition of chamotte had the best mechanical properties before and after thermal exposure (compressive strength up to 95 MPa). The average pore size diameters increased with the increasing temperature (from 10 nm to approx. 700 nm). The fillers addition decreased the pore volume (from 250 mm^3^/g to approx. 100 mm^3^/g).

## 1. Introduction

Alkali activated aluminosilicates, such as inorganic geopolymers, have been attracting attention in the last few years as a new material with a wide variety of application and excellent properties [1,2]. Good mechanical properties, such as resistance to high temperatures and chemicals (especially acids and organic solvents), are the main attributes of geopolymers [3,4,5]. On the basis of their excellent characteristics, various applications have been developed—such as for buildings materials [6], immobilizers of toxic waste [3,7], decorative and restoration materials [8], fiber reinforced geopolymer composites [3,9], coatings [10,11], catalysts [12,13], sorbents [14], and materials for 3D printing [15], to name a few.

Geopolymer binders are formed by mixing powdered aluminosilicates with a liquid alkaline activator. A liquid alkali silicate (water glass) and alkali metal hydroxide solution are usually used as the alkaline activator. As materials rich for Al and Si, there are usually used rice husk ash, demolition wastes, blast furnace slag, volcanic ash, fly ash, or widely used metakaolin (calcined kaolin). During the process of synthesis of geopolymers, the aluminosilicates are partially dissolved in an alkaline medium to a hydrolyzed silicate and aluminate, which are subsequently polycondensed into a three-dimensional polymer network [1,4,16,17,18].

Geopolymer binders are often filled or reinforced by inert solid materials to improve their properties and for the purpose of reducing the price similarly to other binder systems [16,17]. A low viscosity of the geopolymer binder is generally required for good miscibility and possibility of high rate of filling. Previous studies have shown that the preparation of geopolymer binder from calcined kaolinitic claystone and potassium silicate activator resulted in the low viscosity of the binder [17,19]. The type of filler material used can influence phase transformation at a high temperature, and minimize porosity and shrinkage of the material [20]. Various kinds of inert fillers in geopolymer composites—including quartz sand [2,16,21,22,23,24,25], crushed brick [26], corundum [26,27,28], kaolinite [26], chamotte [20,23,25,29], cordierite [30], vermiculite [31], carbon fiber [32,33], or silicon carbide [11]—were investigated in many studies. Buchwald et al. [20] described the influence of fillers on the mechanical properties of geopolymers. They found, among others, that the addition of quartz or chamotte lowered porosity and provided higher compressive strength. Another study documented that the compressive strength of geopolymers increases with the increasing addition of cordierite [30]. Since the geopolymer binders or composites have good resistance to high temperatures, it is desirable to study their properties after heat exposure and at required elevated temperatures (in situ). Barbosa et al. [26,34] studied the thermal behavior of geopolymer binders (sodium or potassium) and their geopolymer composites. They reported that geopolymers synthetized from calcined kaolinite had a relatively stable structure up to 1200 °C. Lemougna et al. [1] tested the influence of an activating solution composition on the stability and thermo-mechanical properties of geopolymers. The geopolymer prepared from the potassium silicate solution promoted thermally more stable material than from the sodium silicate solution. There have been numerous studies which tested the thermo-mechanical properties of geopolymer binders or geopolymer composites after exposure to elevated temperatures (after cooling) [9,22,23,25,28,35]. Nearly no research has been done under real conditions, i.e., at the required elevated temperature (in situ). Martin et al. [36] investigated the effect of temperature (in situ and after exposure) on the mechanical strength of two alkali activated aluminosilicates prepared from fly ash. They discovered that the in situ trials provided comparable results with trials after exposure up to 600 °C temperatures only. 

This work was focused on the effect of the types of fillers on the thermo-mechanical properties of metakaolinite-based geopolymer composites. Four types (sand quartz, chamotte, cordierite, and corundum) of fillers were tested and the results were compared with the properties of the unfilled geopolymer binder. Geopolymer binder was prepared from calcined claystone and potassium alkaline activator.

## 2. Materials and Methods

### 2.1. Materials

**Figure 1:**
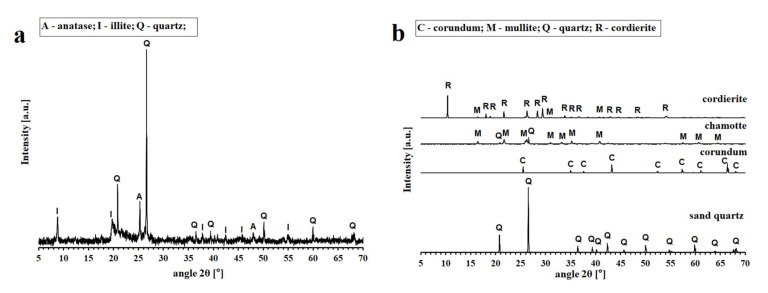
XRD patterns of the aluminosilicate Mefisto L_05_ (**a**) and fillers (**b**).

Raw materials used for the preparation of geopolymer binder were all commercially available products: a metakaolinite-rich material Mefisto L_05_ produced by the calcination of kaolinitic claystone at about 750 °C in a rotary kiln (České lupkové závody, a.s., Nové Strašecí, Czech Republic), potassium silicate (Vodní sklo, a.s., Prague, Czech Republic) and potassium hydroxide pellets (G.R. grade, 88.2 wt % KOH, Lach-Ner, s.r.o., Neratovice, Czech Republic). Sand quartz (Provodínské písky, a.s., Provodín Czech Republic), chamotte (České lupkové závody, a.s., Czech Republic), cordierite (České lupkové závody, a.s., Czech Republic) and corundum (Koltex color s.r.o., Mnichovo Hradiště, Czech Republic) of grain size 0–2 mm were used as natural fillers for the preparation of the geopolymer composites. The chemical compositions of the raw materials are given in Table 1, their physical properties are summarized in Table 2 and the results of X-ray diffraction analysis (XRD, Bruker, Billerica, MA, USA) of aluminosilicate Mefisto L_05_ and fillers are shown in Figure 1.

### 2.2. Preparation of Geopolymers

The alkali activator was obtained by dissolving the solid potassium hydroxide in a commercial potassium silicate solution. The water absorbed during milling and storage was removed from the metakaolinite component Mefisto L_05_ by drying at 110 °C. The geopolymer binder was synthesized by stirring the metakaolinite component with an alkali activator in a planetary mixer at room temperature for 10 min. Distilled water was then added to achieve a total water content of 30% in the geopolymer binder and stirring continued for another 5 min. The mixtures were poured into silicon molds and placed on the vibration table for 5 min in order to remove air bubbles. The molds were sealed into polyethylene bags and cured at 60 °C for 4 h in an electric oven. The samples were de-molded and left to cure at room temperature (20 °C) for 7 days. The curing conditions, including time, were chosen on the basis of the results of Rovnaník et al. [37], which verified that, under these conditions, the samples reached final strengths.

The prepared geopolymer binder had molar ratios of Me:Al 1, Si:Al 1.5, Me:Si 0.66 and a total water content of 30%. The Me indicates an alkali metal, total water content involved water contained in commercial potassium silicate solution and potassium hydroxide just as that added during the binder preparation. The geopolymer binder was chosen on the results of our previous works, where the geopolymer binders based on Mefisto L_05_ provided binders with very low viscosity and excellent mechanical properties [17,19]. The geopolymer binder was labeled as GB. 

Geopolymer composites containing 65 vol % of different granular inorganic fillers were prepared by adding sand quartz, corundum, chamotte, or cordierite to pure geopolymer binder (GB) and mixed for another 5 min. The curing conditions were the same as for the geopolymer binder. These composites were labeled GQ, GC, GS, and GR, in analogy to geopolymer composites with sand quartz, corundum, chamotte, or cordierite.

Samples of geopolymer binder and geopolymer composites cured for 7 days as described above, were exposed to heating at 200, 400, 600, 800, 1000, and 1200 °C. The samples were placed into an electric furnace (Clasic CZ, type 5013V, Řevnice, Czech Republic) and heated at a rate of 5 °C/min, and maintained for 1 h at each desired temperature. Subsequently, the samples were cooled inside the furnace down to room temperature. The samples were labeled as GX-Y, where X indicated geopolymer binder or composite and Y indicated heat exposure in °C.

### 2.3. Analytical and Testing Methods

Chemical compositions of the solid raw materials were determined by X-ray fluorescence (XRF, Bruker, Billerica, MA, USA) using a BRUKER S8 Tiger instrument.

Particle size distribution of the aluminosilicate Mefisto L_05_ was measured by a Mastersizer 2000 laser diffraction particle size analyzer (MALVERN Instruments, Malvern, UK). Agglomerates were disrupted by ultrasound treatment.

A pycnometric method was used to determine specific gravity and bulk density by pouring powder into a cylindrical vessel.

Brunauer–Emmett–Teller (BET) surface area of the aluminosilicate Mefisto L_05_ was determined by nitrogen adsorption using an Autosorb iQ (Quantochrome Instruments, Boynton Beach, FL, USA).

An AutoPore IV 9510 mercury intrusion porosimeter (Micromeritics, Unterschleißheim, Germany) was used to determine the pore size distributions of geopolymer binder and geopolymer composites before and after exposure to high temperature up to 1200 °C. The porosimeter operates with pressures from 0.01 MPa to 414 MPa. Pore size distribution was evaluated in the range of 4–350,000 nm for geopolymer binders and composites (full measurement range). 

An inductively coupled plasma optical emission spectrometer (ICP-OES) OPTIMA 8000 (Perkin Elmer, Waltham, MA, USA) was used to determine the content of micro-elements and the K/Na ratio in the liquid potassium silicate. Conventional acid–base titration methods were used for determining total content of alkali metals (Na, K) and SiO_2_ in the potassium silicate (module) solutions. These methods were preferred for better accuracy at higher concentrations.

The phase composition of the clay material was identified by means of a BRUKER D8 Advanced X-ray diffraction system (XRD) equipped with a BRUKER SSD 160 detector and operating with Cu-Kα radiation at 40 kV and 25 mA. XRD scanning was taken at the step 2θ = 0.02 over an angular range from 5° to 70° with 1 s counting time. The phase composition of the geopolymer binder and composites tested at in situ temperatures from 30 °C to 1200 °C were identified by means of a BRUKER D8 Advanced X-ray diffraction system (XRD). The instrument was equipped with a high-temperature oven chamber during measurement. The temperature was increased at a rate of 5 °C/min and lasted for 1 h at each desired measured temperature. The samples were measured on a plate of platinum.

A universal testing machine, LabTest 6.200 (Labortech, Opava, Czech Republic) fitted with an electric furnace, allowing testing of mechanical properties up to 1200 °C was used for the determination of mechanical properties. Flexural strength was determined using a three-point-bending test on six samples (20 × 20 × 160 mm) of geopolymer binder and geopolymer composites before and after exposure to high temperature up to 1200 °C with a crosshead speed of 0.1 MPa/s (approx. 0.25 mm/min). Compressive strength and modulus of elasticity were measured according to the ISO 1920-10 standard on six prismatic samples (30 × 30 × 64 mm) of geopolymer binder and geopolymer composites before and after exposure to high temperature up to 1200 °C with a crosshead speed of 0.5 MPa/s (approx. 0.25 mm/min). The compressive strength and modulus of elasticity of the geopolymer binder and composites tested in vivo at temperatures from 25 °C to 1200 °C were also tested. The compressive strength and modulus of elasticity of the geopolymer binder and composites were measured on the three prismatic samples (30 × 30 × 64 mm). The temperature was increased at a rate of 5 °C/min and lasted for 1 h at each desired measured temperature. Mechanical properties were determined 7 days after the sample preparation. 

Dilation tests up to 1200 °C using a dilatometer (Clasic CZ, type DIL 1500, Řevnice, Czech Republic) were carried out on 5 × 5 × 50 mm samples of geopolymer binders and 20 × 20 × 160 mm of geopolymer composites. The measurements were conducted under the same heating conditions (5 °C/min) for all the samples.

A testing machine for the determination of refractoriness under load (Clasic CZ, type ZARO 17, Řevnice, Czech Republic) of geopolymer binders and geopolymer composites was used. The refractoriness under load was determined according to the Czech standard CSN 993-8. This test was carried out with a hollow cylinder having the dimensions of 5 × 5 cm. There was a circular hole with a diameter of 12 mm in the center of the cylindrical sample. The temperature increased at a rate of 5 °C/min and the loading applied on the sample amounted to 0.2 MPa.

A heat microscope for the determination of pyrometric cone refractoriness (Clasic CZ, type 0116 VAK, Řevnice, Czech Republic) of geopolymer binders and geopolymer composites was used. Pyrometric cone refractoriness was determined according to the European standard EN 993-12. This test was carried out under atmospheric pressure on three samples with a sharp-edged, trilateral, oblique, truncated cone having the dimensions of 30 × 8.5 mm. The temperature was increased at a rate of 5 °C/min. During heating, the tested samples were observed and compared with the pyrometric reference cones. The refractoriness of the tested samples is considered as between the nearest two reference cones that melt simultaneously with the tested one.

## 3. Results and Discussion

### 3.1. Phase Composition

**Figure 2:**
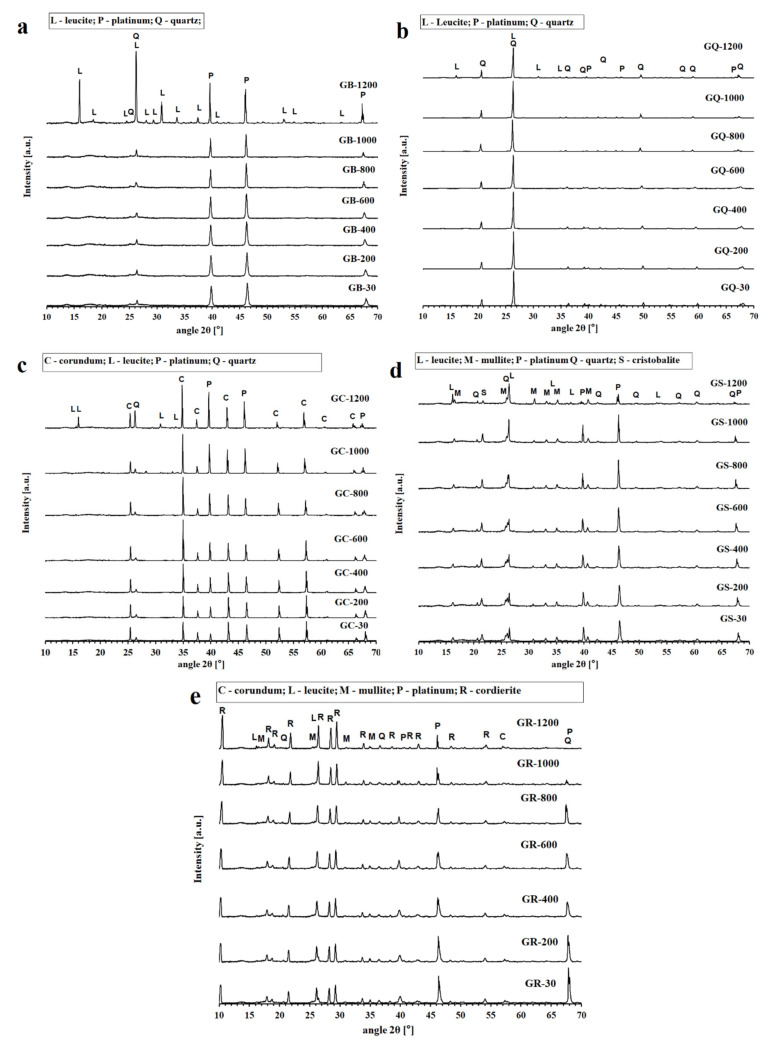
XRD patterns of geopolymer binder (**a**) and geopolymer composites (**b**—quartz, **c**—corundum, **d**—chamotte, **e**—cordierite) tested in situ at temperatures from 30 °C to 1200 °C.

The XRD patterns of the geopolymer binder and geopolymer composites tested in situ at temperatures from 30 °C to 1200 °C, are reproduced in Figure 2. The impurities of platinum in all examined samples was due the plate of platinum, which was part of the device. The X-ray patterns of the geopolymer binder (Figure 2a) did not reveal any changes in amorphous phases up to 1000 °C. From 1000 °C up to 1200 °C the XRD spectra revealed the presence of crystallized leucite (KAlSi_2_O_6_) as the main phase. Leucite was also observed in geopolymer binder prepared from metakaolin and potassium alkaline activator after heat exposure at 800 °C [38], 1000 °C [34,39], 1050 °C [40], and 1300 °C [11].

Quartz was a quite inert phase that does not react with the geopolymer matrix; therefore, no differences between the XRD patterns of GB-30 (tested at room temperature) and GQ-1000 (tested in situ at temperature 1000 °C) were observed. Changes in the XRD pattern were captured after the exposure to 1200 °C, when phases of leucite occurred. Similar results (presence of leucite) of geopolymer composites with quartz after heat exposure were observed by Kamseu et al. [27]. XRD results of GC, GS, and GR samples follow the same path as a GQ samples. Phase of leucite were detected in GC, GS, and GR samples after exposure to 1000 °C. Lin et al. [38] observed the presence of crystalline leucite in the prepared geopolymer composites with the addition of corundum after heat exposure to 1000 °C. Mullite (Al_6_Si_2_O_13_) is the main component of chamotte as shown in Figure 1b. Similarly, the cordierite (Mg_2_Al_4_Si_5_O_18_) is the main component of cordierite.

The samples after exposure to high temperature were also examined by XRD method. The results are not part of this publication because the results were comparable with the diffraction results tested in situ. The only difference was the detection of a new phase (leucite) in GB sample already after exposure to 800 °C. The difference may be caused due to the absence of cooling process in the case of in situ sample testing.

### 3.2. Thermal Properties

#### 3.2.1. Thermal Dilatometry

**Figure 3:**
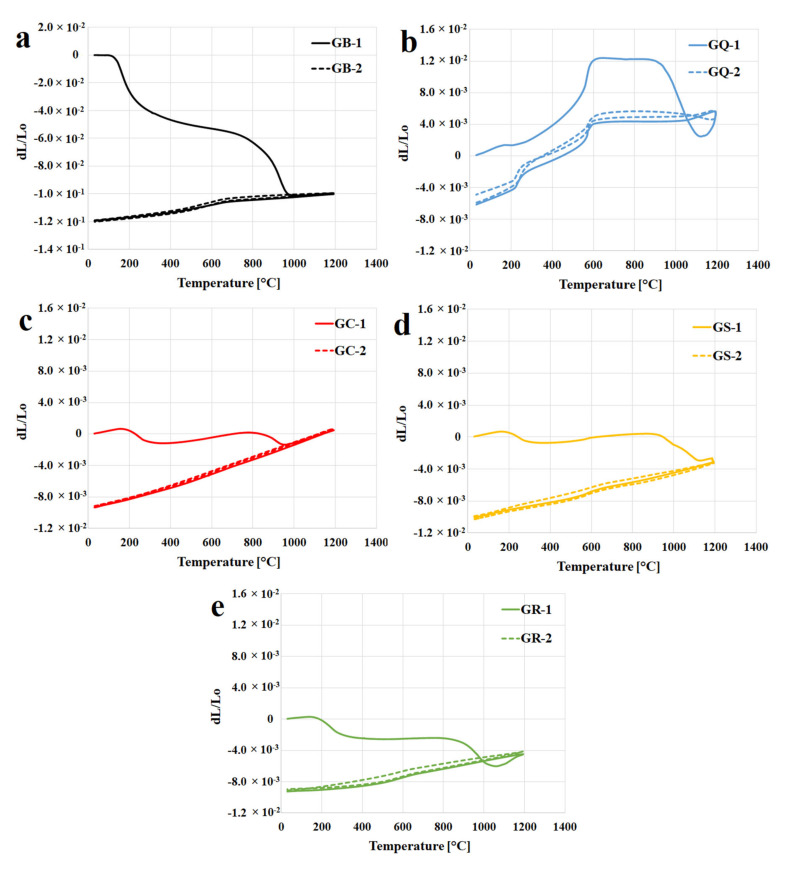
The dilatometric curves of first and second runs of geopolymer binder (**a**) and composites (**b**—quartz, **c**—corundum, **d**—chamotte, **e**—cordierite) up to 1200 °C (5 °C/min).

Figure 3a displays the dilatometric curves (first and second run) of geopolymer binders measured from about 30 °C to 1200 °C with a heating rate of 5 °C/min. The curve of dilatation showed that there was significant shrinkage of geopolymer binder from the temperature of 150 °C to 900 °C. The changes in the structure of the geopolymer binder were the reasons for shrinkage during heating. The geopolymer binder started to shrink due to dehydration of free water from the pores up to 300 °C. The main reason for the shrinkage in the temperature region from 300 °C to 900 °C was the dehydroxylation. Above 900 °C, sample of GB started to expand slightly up to the temperature limit of the experiment (1200 °C). This was probably caused as a result of crystallization of a new phase (leucite). These claims are in good agreement with the published results of TGA analysis of potassium geopolymer binders previously published by other authors [24,38,41]. During cooling, the geopolymer binder shrank more or less linearly. Over the second run of dilatometry, the geopolymer binder expanded linearly. Thus, when the samples cooled, the remaining change of length is attributed to recovery of the thermal expansion. Cooling produced comparable curves as in the first run of cooling, which is in agreement with other studies [1,21]. The total shrinkage of geopolymer binder was about 10% over its total length after the first run of heating and cooling. The total shrinkage of potassium geopolymer binder (GB) was similar or lower in comparison with the works of Kuenzel et al. [42] (total shrinkage by 25%), Kovařík et al. [29] (total shrinkage by 15%), and Barbosa et al. [26] (total shrinkage by 9%). Differences may be caused by a different geopolymer binder composition. The linear coefficient of thermal expansion (α) of the GB was about 2.6 × 10^−5^/°C, the value was measured between 200 and 600 °C during the second run of heating.

Dilatations (first and second run) of the geopolymer composites are shown in Figure 3b–e. The measured data suggested that the thermal changes of geopolymer composites represent a combination of the properties of the geopolymer binder and the fillers. Linear coefficients of thermal expansion of the quartz, corundum, chamotte, and cordierite are about 7.9, 4.8, 4.5, and 5.7 × 10^−6^/°C, the values were measured in the temperature range from 25 °C to 900 °C [43,44,45,46]. 

The curves show that all experimental samples of geopolymer composites in the first run exhibited small expansion from laboratory temperatures up to 150 °C, probably due to expansion of the fillers. An almost flat part in the thermal dilatometry curves of all the composites (excluding GQ) from 150 °C up to about 900 °C indicate a useful working temperature range. Geopolymer containing quartz as a filler demonstrated an abrupt expansion from 200 °C to 600 °C followed by a dimensionally stable phase up to 900 °C. The abrupt thermal expansion of quartz is connected to quartz transition at 573 °C (from the room temperature stable alpha phase to the beta phase) [25]. The presence of fillers was found to significantly reduce the shrinkage. The optimal behavior under heating was exhibited by the samples with the addition of corundum or chamotte. The total shrinkage of all geopolymer composites was very low and did not exceed 1% over its total length after the first run of heating and cooling. Similar shrinkage of metakaolin geopolymer binder with chamotte as an aggregate was described by Rovnaník et al. [47]. The total shrinkage of their sample also did not exceed 1%.

The second run of dilatometry of geopolymer composites (excluding GQ) had the same pattern as the geopolymer binder, with the samples expanding linearly. Nonlinearity of GQ was due to the reversibility of the transition between the two forms of quartz. The linear coefficients of thermal expansion (α) of the GQ, GC, GS, and GR were about 22.1, 8.5, 6.4, and 5.0 × 10^−6^/°C, where the values were measured between 200 °C and 600 °C during the second run of heating.

#### 3.2.2. Pyrometric Cone Refractoriness

**Figure 4:**
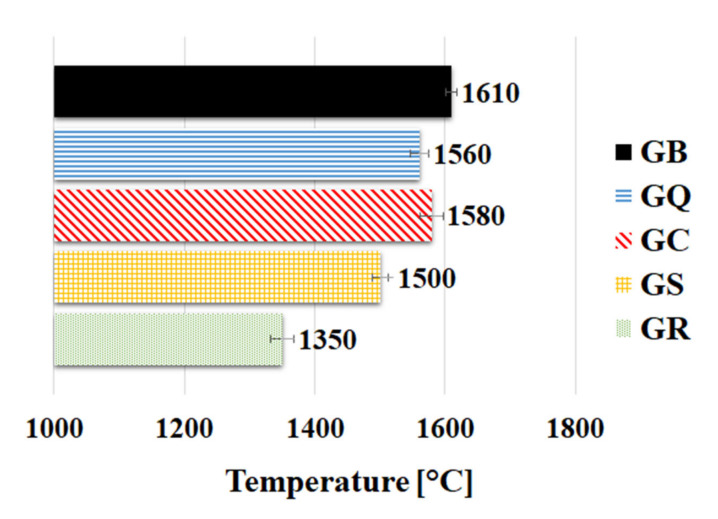
Pyrometric cone refractoriness of geopolymer binder (GB) and composites (GQ—quartz, GC—corundum, GS—chamotte, GR—cordierite).

The results of refractoriness for the geopolymer binder and composites are shown in Figure 4. The determined pyrometric cones refractoriness of geopolymer binder and composites was expressed by the results of pyrometric reference cones that melted simultaneously with the tested sample. From the result of refractoriness of geopolymer binder (GB), it is evident that the binder can withstand high temperatures of up to 1610 °C (melting does not occur before this temperature). Application of the fillers into the geopolymer binders reduces their refractoriness. The decrease in refractoriness may be due to earlier sintering of filler and the geopolymer matrix. The results indicated different interphase processes during sintering of binder compared to geopolymer composites. Geopolymer composites with the addition of sand (GQ) or corundum (GC) experienced only a slight decrease in refractoriness compared to GB. The biggest drop of refractoriness value was observed in the geopolymer composite with the addition of cordierite (GR). The drop in refractoriness of GR may be related to the melting point of the filler. The melting point of cordierite is around 1400 °C, depending on the condition [48]. The best refractoriness of geopolymer composite was achieved with corundum (1580 °C) followed by quartz (1560 °C), chamotte (1500 °C), and lastly cordierite (1350 °C). According to ASTM C71 [49], the geopolymer binder and composites presented in this study can be considered as refractory material.

#### 3.2.3. Refractoriness Under Load

**Figure 5:**
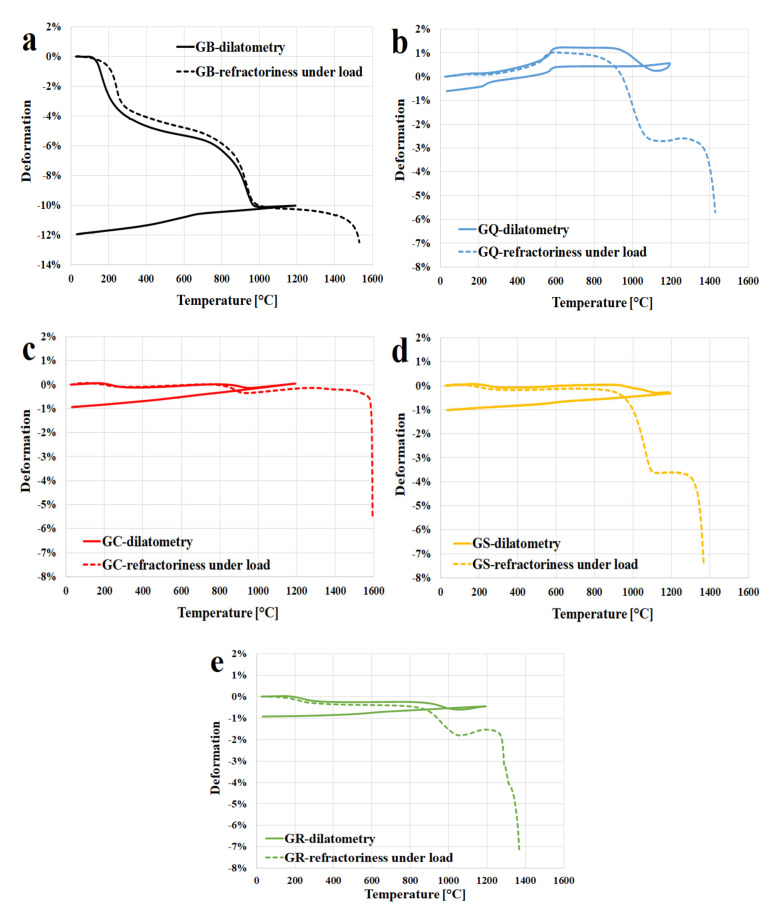
Comparison of refractoriness under load in relation to dilatometry geopolymer binder (**a**) and composites (**b**—quartz, **c**—corundum, **d**—chamotte, **e**—cordierite).

Figure 5 represents the results of comparison of refractoriness under load in relation to dilatometry of geopolymer binder (Figure 5a) and composites (Figure 5b—quartz; Figure 5c—corundum; Figure 5d—chamotte; Figure 5e—cordierite). According to the standard, Figure 5 shows a dependence of relative deflection on the temperature, temperatures T0.5 and T5 represent temperatures at which 0.5% and 5% deformation of the cylinder will occur against its maximum height (height also changes due to thermal expansion). The determination of the refractoriness under load of GB (Figure 5a) displayed that the T0.5 and T5 values were obtained for temperatures at 200 °C and 600 °C. This rapid decrease is probably due to dehydroxylation as it was described in dilatometry. From the comparison, it was evident that the curves of refractoriness under load and dilatometry were the same up to 1200 °C (the temperature limit of the dilatometry). Therefore, the refractoriness under load did not affect the shape changes of geopolymer binder up to 1200 °C, but the dilatation was measured. 

The temperature of deformation of geopolymer binder increases with addition of fillers as indicated by Figure 5b–e. The addition of quartz (Figure 5b), chamotte (Figure 5d) or cordierite (Figure 5e) increase the T0.5 temperature up to 1000 °C and T5 up to 1400 °C compared to GB. The geopolymer composite with the addition of corundum (Figure 5c) had the best result of refractoriness under load. The value of T0.5 temperature of GC was also around 900 °C, but the T5 temperature was almost at 1600 °C. Previous study found out that with increasing corundum content, the temperature of deformation of fire clay refectories increases [50]. The results of refractoriness under load of geopolymer composites clearly illustrate two breaks in the curve. The first break (around 1000 °C for all geopolymer composites) is probably caused by the crystallization of a new crystalline phase. This claim is consistent with the results of XRD analysis. The second break (between 1400 °C and 1600 °C) can be due to melting of geopolymer materials, which also agrees with the results of pyrometric cone refractoriness. From the comparison of refractoriness under load in relation to dilatometry of geopolymer composites, it emerged that the curve of refractoriness under load had the same progress as the dilatometry curve up to temperature 1000 °C. There were clearly visible differences between the curves from the temperature of 1000 °C. From this temperature, the refractoriness under load was already measured.

### 3.3. Mechanical Properties

**Figure 6:**
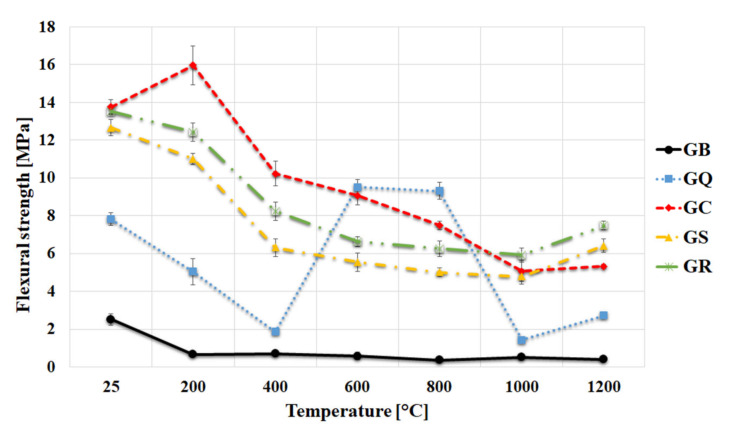
Flexural strength of the geopolymer binder and geopolymer composites at room temperature after exposure up to 1200 °C.

Average values of flexural strength of geopolymer binder and geopolymer composites before and after heat exposure are depicted in Figure 6. The addition of fillers had a significantly positive effect on the flexural strengths. Geopolymer composites with the quartz, corundum, chamotte, and cordierite as fillers exhibited much higher flexural strength than the corresponding geopolymer binder. The changes in flexural strengths after exposure to increasing temperatures followed the decreasing path up to 1000 °C. The heating temperature of 1200 °C resulted in a slight increase in flexural strength. This finding may be explained by the changes in phase composition (formation of leucite). The cause of the drop in flexural strength of GB during exposure to high temperature was probably due to the dehydration and internal cracking of GB during heating [29]. A similar drop was also observed for GC, GS, and GR after heating to 400 °C, but the drop in the flexural strength was not so big because the fillers are able to alleviate the flexural stress caused by shrinkage of the GB. A large increase in flexural strength was observed after heating the geopolymer composite filled with quartz to 600 °C and 800 °C. This can be interpreted as a result of phase inversion of quartz at 573 °C (from the room temperature stable alpha phase to the beta phase) [25]. 

The geopolymer binder had low flexural strength values at either room temperature (2.5 MPa) or elevated temperature (1200 °C—0.5 MPa). Kuenzel et al. [42] also examined the potassium geopolymer binder and reached an analogous value of flexural strength at laboratory temperature (3 MPa). Samples with corundum (13.8 MPa), chamotte (12.7 MPa), and cordierite (13.5 MPa) reached the highest flexural strength at room temperature. The flexural strength of the sample with quartz was noticeably lower (7.9 MPa) at room temperature. Samples with corundum, chamotte and cordierite retained good values of flexural strength (GC—5.3 MPa, GS—6.4 MPa, and GR—7.5 MPa), even after exposure to 1200 °C. Musil et al. [51] reached similar results of flexural strength of geopolymer composites with addition of chamotte at room temperature (13 MPa). However, the values after thermal exposure were lower (1200 °C—3.44 MPa). Lower values can be caused by a lower addition of chamotte aggregate or that the testing of mechanical properties was conducted in situ.

**Figure 7:**
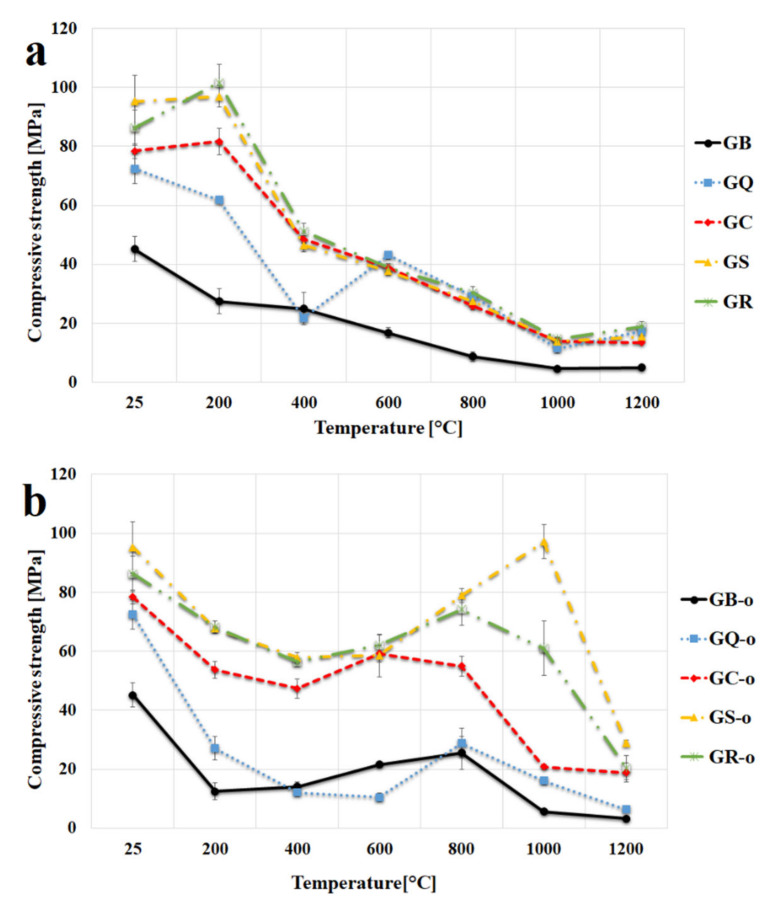
Compressive strength of the geopolymer binder and geopolymer composites at room temperature and after exposure up to 1200 °C (**a**) and in situ temperature from 25 °C to 1200 °C (**b**).

The effect of heat exposure up to 1200 °C on the compressive strength of the prepared materials is demonstrated in Figure 7a. The results confirmed the earlier published finding, i.e., significant decrease in compressive strength with increasing temperature (from about 45 MPa to about 5 MPa at 1000 °C) in geopolymer binders [36,52,53,54,55]. The reduction in strength with rising temperature is probably due to the dehydration of the geopolymer matrix, weakening the structure, and also to the possible concomitant development of surface cracks and internal damage in the overall structure of the geopolymer [21]. Therefore, the low results of compressive strength of geopolymer binder are due to high shrinkage after heating (see Figure 4). Kovařík et al. [29] reported the equivalent values of compressive strength of potassium geopolymer binder at the laboratory temperature (40 MPa) and even at elevated temperature of 1000 °C (8 MPa).

As could be expected, the addition of fillers had a beneficial effect on the mechanical properties of geopolymer composites either at laboratory temperature or an elevated temperature. The addition of fillers mitigates the shrinkage of the geopolymer binders (the material does not crack due to heating). The results indicate decreases in compressive strength of geopolymer composites with increasing temperature, which were also previously published [23,25,30,47,56]. A slight increase in compressive strength after exposure to 200 °C was observed with GC, GS, and GR samples. It seems, in view of previous research results [47], the strengthening of the materials can be attributed to the stiffening of the gel and the increase in surface forces between the particles due to the release of adsorbed moisture or the promotion of polycondensation between chain-like geopolymer gels. The exposure to 1200 °C also increased the compressive strength of geopolymer composites. Similarly to flexural strength, the increase was probably due to the formation of a new crystalline phase of leucite. A reduction in compressive strength was observed with the sample of GQ after exposure to 400 °C. The deterioration in compressive strength is possibly due to the change of quartz from the stable alpha phase to the beta phase [25]. 

The highest compressive strength at room temperature was achieved with the sample with the addition of chamotte (95.2 MPa), followed by cordierite (86.3 MPa), corundum (78.4 MPa) and lastly quartz (72.4 MPa). All examined geopolymer composites had comparable results of compressive strength after calcination to 600 °C and higher (approx. 40 MPa at 600 °C, approx. 30 MPa at 800 °C, approx. 14 MPa at 1000 °C, approx. 19 MPa at 1200 °C). 

Measured values of compressive strength of geopolymer composites at laboratory temperature and even after the exposure to a higher temperature could be considered as high compared to the previously published results of compressive strength of metakaolinite-based geopolymer composites. For example, Sarkar et al. [57] tested the geopolymer composite based on metakaolin and sodium activator with the addition of corundum (50 wt %) and the compressive strength was 35 MPa at laboratory temperature. Hemra et al. [30] examined the geopolymer composite based on metakaolin and sodium activator with the addition of cordierite (30 wt %) and the compressive strength was 57.5 MPa at room temperature. Rovnaník et al. [25,47] studied the geopolymer composite based on metakaolin and sodium activator with the addition of chamotte (58 wt %) and the results of compressive strength were 37 MPa at 25 °C, 50 MPa at 200 °C, 29 MPa at 400 °C, 27 MPa at 600 °C, 15 MPa at 800 °C, 8 MPa at 1000 °C, and 10 MPa at 1200 °C. Trindadea et al. [23] also revealed a descending paths of compressive strength of geopolymer composites based on metakaolin and sodium activator with the addition of chamotte or quartz (50 wt %) with increasing temperature. The described results of compressive strength of geopolymer composites were 72 MPa for quartz and 62 MPa for chamotte at laboratory temperature and 7 MPa for quartz and 18 MPa for chamotte after the exposure to 1000 °C. The reason for the high values of compressive strength of the prepared geopolymer composites was probably the appropriate binder composition, especially its low viscosity, which made it possible to achieve a high fill rate (high content of filler in the composite) and thus excellent mechanical properties after thermal exposure.

Most published results are derived from ex situ experiments (away from the heating environment). This paper is also focused on the results from in situ experiments (testing in the heating environment). The compressive strength of tested samples at the required temperature from 25 °C to 1200 °C is shown on Figure 7b. Results of compressive strength of geopolymer binder and geopolymer composites tested in situ did not follow the same decreasing path with the increasing temperature as it was described above (via Figure 7a). In situ compressive strength of examined materials started to decrease with increasing temperature up to 400 °C, the strength was lost due to previously mentioned dehydration. However, from 600 °C the strength of prepared materials increases up to 800 °C. The reasons for the increase in strength after 400 °C are not yet known, but it could be due to the absence of a cooling process during in situ measurement. Therefore, the samples tested in situ did not shrink compared to samples tested after exposure to high temperature (see Figure 3). A drop in compressive strength occurred after heating to 1000 °C and it was probably ascribed to the previously mentioned crystallization of a new phase. GS provided a remarkably result of strength (97.1 MPa) at 1000 °C temperature, which was comparable with strength at ambient temperature (95.2 MPa). Better bonding of the geopolymer binder to the porous chamotte or the plastic-like behavior of the sample could cause a high increase in compressive strength.

**Figure 8:**
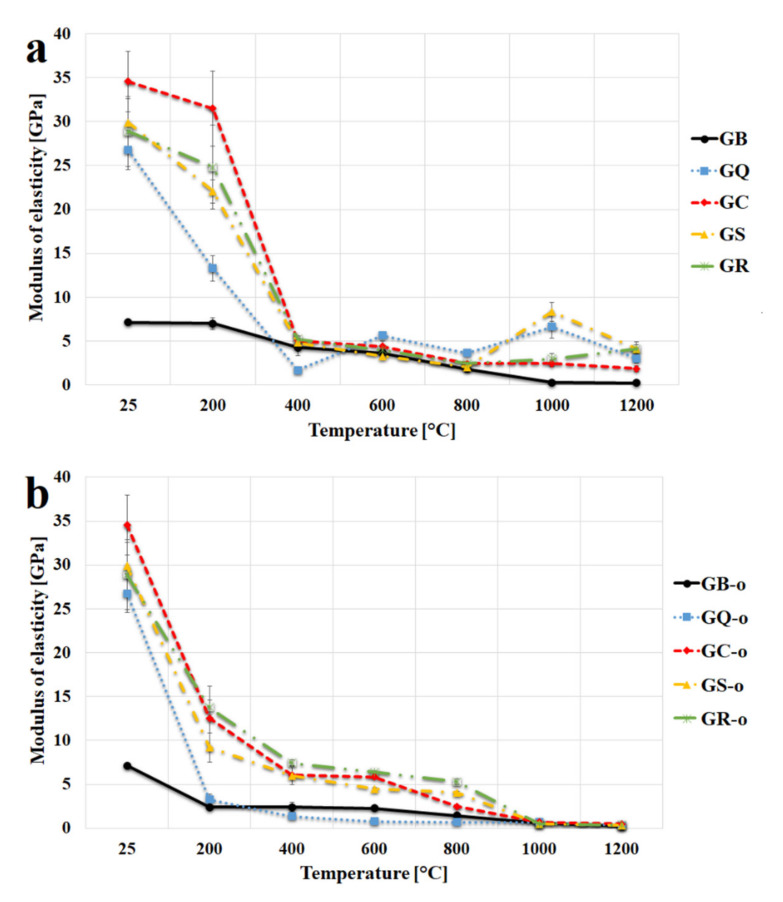
Modulus of elasticity of the geopolymer binder and geopolymer composites at a room temperature after exposure up to 1200 °C (**a**) and in situ at temperatures from 25 °C to 1200 °C (**b**).

The modulus of elasticity of geopolymer binder and composites after temperature exposure up to 1200 °C is shown in Figure 8a. The trends of elastic moduli observed in geopolymer binders and composites after calcination followed quite similar trends of compressive strength (Figure 7a), which implies a decrease in modulus of elasticity with higher temperature and significantly higher values for geopolymer composites compared to geopolymer binder. The stiffness of geopolymer composites was greatly improved due to the low compressibility of the filler. From 200 °C, the samples showed a significant drop in the modulus of elasticity. Reduction of the modulus of elasticity was caused by dehydration from the pores in the matrix.

The geopolymer binder had an elasticity modulus of 7.2 GPa at room temperature and 0.3 GPa after exposure to 1200 °C. The modulus of elasticity in the prepared geopolymer binder was consistent with the previously published results (5–7.8 GPa) [58,59,60]. The highest elasticity modulus at laboratory temperature was found in the sample with the corundum content (34.6 GPa), followed by chamotte (29.9 GPa), cordierite (28.9 GPa), and quartz (26.8 GPa). The actual moduli in the prepared geopolymer composites varied between 1.9 to 4.1 GPa after calcination at a high temperature (1200 °C). Rocha et al. [24] mechanically tested the potassium geopolymer composite with the addition of quartz and the modulus of elasticity was lower (7.7 GPa) than in this study. The lower value of modulus of elasticity could be due to different chemical composition (Si/Al) or different ratio of binder/filler (mass ratio was 1:1). 

**Figure 9:**
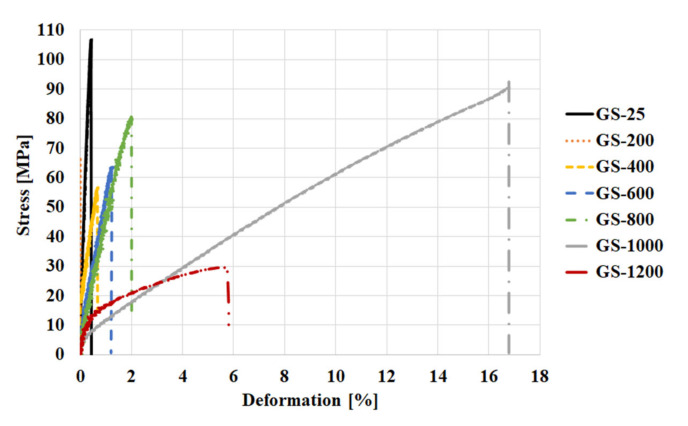
In situ compression test stress–deformation curves at different temperatures for chamotte geopolymer (GS).

Figure 8b presents the modulus of elasticity of geopolymer binder and composites tested in situ at temperature from 25 °C to 1200 °C. The tendency of the modulus of elasticity in situ is to track the same decreasing path with increasing temperature as in Figure 8a. The modulus of elasticity of tested materials was almost zero after heated up to 1000 °C and 1200 °C. The samples had more plastic-like behavior with the higher temperature as shown in the Figure 9. Figure 9 displays the curves of stress-deformation of GS samples tested in situ at different temperatures. The presented curves were selected as the most representative out of the six measured curves at each examined temperature. Figure 9 shows only the GS sample because similar dependences applies to the other samples. According to these curves the GS exhibited a linear elastic behavior from laboratory temperature up to 800 °C. At higher temperatures, from 1000 °C to 1200 °C, the behavior of GS was plastic. These results are in agreement with previously published results, which exhibited similar curves of geopolymer prepared from fly ash [36]. The plastic behavior of geopolymer composites at high temperature was also shown in Figure 5 (refractoriness under load), where the crystallization of new phases from 1000 °C caused the deformation of tested materials under load.

### 3.4. Porosity

**Figure 10:**
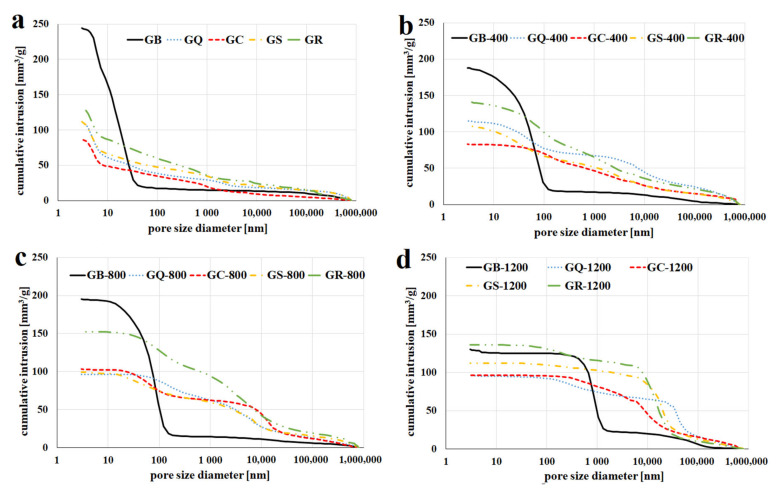
Mercury intrusion porosimetry of the geopolymer binder and geopolymer composites at room temperature (**a**) and after exposure to 400 (**b**), 800 (**c**), and 1200 °C (**d**).

Mercury intrusion porosimetry was used to determine the pore size distribution in geopolymer binder and composites after hardening at an ambient temperature and after heat exposure. Figure 10 shows the cumulative intrusion volume of geopolymer binder and composites at a laboratory temperature (Figure 10a) and after exposure to 400 (Figure 10b), 800 (Figure 10c), and 1200 °C (Figure 10d). The GB contained capillary pores larger than 100 nm and gel pores smaller than 10 nm after hardening at laboratory temperature. It can be seen that the pore volume rapidly dropped (from 250 to 130 mm^3^/g) and the pore size increased (especially from a temperature above 800 °C) due to the shrinkage and crystallization of new phases in the geopolymer matrix with elevated temperature in the GB. Average pore size diameter rose from 10 nm (25 °C) to 100 nm (1200 °C). The results correlate to some extent with a previous study which measured the pore size distribution of geopolymer binder prepared from metakaolin and sodium alkaline activator [47]. They found out that the pore volume was 280 mm^3^/g at room temperature and dropped to 40 mm^3^/g at the temperature of 1000 °C. 

The use of fillers caused a decline in pore volume of geopolymer composites compared to GB at room temperature (from 250 mm^3^/g to approx. 100 mm^3^/g). As the calcination temperature increased, the pore volume of the geopolymer composites did not change significantly, unlike the geopolymer binder. While the pore volume of the GB decreased by 48% due to heating at 1200 °C. The highest drop for geopolymer composites was only 12% for the sample of GC. The effect of high temperature exposure on the pore size of geopolymer composites had the same effect as on the GB (the pore size increased). Occurrence of the large pores after heat exposure can be ascribed to the crystallization of the new phases which causes the shrinkage of the geopolymer matrix. 

The smallest pore volume during the measurement was observed in the sample filled with corundum (in the range of 70–100 mm^3^/g), followed by quartz (in the range of 100–110 mm^3^/g), chamotte (in the range of 100–113 mm^3^/g) and lastly cordierite (130–150 mm^3^/g). The geopolymer composite with the addition of quartz had the lowest values of average pore size diameter (102 nm) after heat exposure to 1000 °C, followed by chamotte (228 nm), cordierite (430 nm), and corundum (909 nm). The differences in porosimetry of geopolymer composites were due to different porosity and sintering temperatures of the fillers. Rovaník et al. [25] had slightly lower or even results of pore size distribution of geopolymer composites (quartz or chamotte) prepared from slag and sodium alkaline activator. The values of pore volume of geopolymer filled with quartz (chamotte) were 100 (90) and 110 (75) mm^3^/g after calcinating to 600 °C and 1200 °C.

### 3.5. Morphology

**Figure 11:**
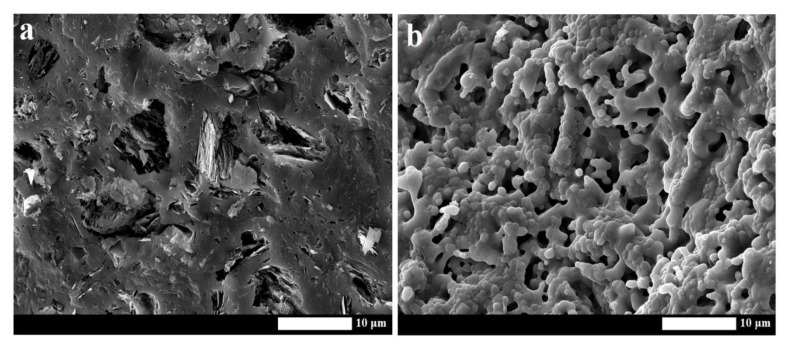
Micrographs of geopolymer binder at room temperature (**a**) and after exposure to 1200 °C (**b**).

The changes in structure observed by scanning electron microscope are shown in Figure 11. This figure clearly illustrates the differences between the structures of the geopolymer binder before and after exposure to high temperature. The surface of the samples at a laboratory temperature (Figure 11a) and after exposure to 1200 °C (Figure 11b) is presented at micrographs. Amorphous geopolymer matrix in geopolymer binder or composites contained a large number of undissolved plates of metakaolinite at an ambient temperature after hardening. The structure was inhomogeneous and did not contain a large number of visible pores. When the material was heated, the morphology of geopolymer matrix rapidly changed due to phase changes. The structure was homogeneous and porous with a pore size in the order of hundreds of nm. These findings are consistent with the previous works [39,40], which documented the behavior at elevated temperature of the geopolymer binders prepared from metakaolin and potassium alkaline activator.

## 4. Conclusions

This paper presents an investigation of the thermo-mechanical behavior of geopolymer binder prepared from calcined kaolinitic claystone and potassium silicate activator, which is characterized by low viscosity and geopolymer composites with different type of fillers (quartz, corundum, chamotte, and cordierite). The following conclusions have been drawn from the obtained experimental results:

- Geopolymer binder based on calcined claystone and potassium alkaline activator had satisfactory workability for the incorporation of a wide content (65 vol %) of different fillers.
- The X-ray patterns of examined samples showed stable amorphous phase up to 1000 °C. XRD spectra changed after thermal treatment between 1000 °C and 1200 °C due to the crystallization of a new phase (leucite).
- The geopolymer binder represents a material with a high thermal shrinkage during exposure up to 1200 °C. The presence of all examined fillers (quartz, corundum, chamotte, cordierite) reduced the shrinkage of geopolymer and increase the temperature of deformation (T0.5, T5). On the contrary, the geopolymer composites had lower temperatures of refractoriness.
- The mechanical properties significantly decreased with increasing temperature. The hardened geopolymer binder exhibited much worse mechanical properties than geopolymer composites. The best mechanical properties were seen in the samples prepared with addition of chamotte and cordierite.
- The results of compressive strength of geopolymer binder and geopolymer composites tested in situ did not match the results of compressive strength of geopolymer binder and composites after exposure to high temperature. The results of compressive strength tested in situ started to increase from 400 °C.
- The trends of modulus of elasticity observed in geopolymer binders and composites followed quite similar trends as compressive strength.
- The values of modulus of elasticity tested in situ decreased with rising temperature, however after heating up to 1000 °C they were almost zero. Their plastic behavior at higher temperatures was the main reason for low values of modulus of elasticity.
- Average pore diameter increased with increasing temperature in the geopolymer binder and composites. The addition of fillers lowered the pore volume.

The results of this study have shown that geopolymer composites filled with corundum or chamotte had the most optimal properties. GC and GS exhibited the lowest shrinkage initially with heating, high refractoriness, and high strength at laboratory temperatures, after thermal exposure and in situ under high temperature. The working temperature of these composites under load was up to 900 °C. The geopolymer composites without the application of load could withstand temperatures up to 1500 °C (composite with chamotte), and respectively 1580 °C (composite with corundum). The low viscosity of the tested binder enabled a higher filling rate, which resulted in high mechanical properties. The geopolymer composites based on calcined kaolinitic claystone and filled with corundum or chamotte can be used as an alternative refractory material due to their excellent thermo-mechanical properties.

## Figures and Tables

**Table 1:** Chemical composition (wt %) of raw materials.

Material	Material Composition (%)											
	^a^ LOI	H_2_O	SiO_2_	Al_2_O_3_	Fe_2_O_3_	CaO	MgO	Na_2_O	K_2_O	TiO_2_	P_2_O_5_	ZrO_2_
Mefisto L_05_	1.18	-	52.1	42.8	0.91	0.18	0.15	0.05	0.77	1.61	0.07	0.03
Potassium silicate	-	60.4	27.0	0.05	0.01	-	-	0.38	12.1	-	-	-
Quartz sand	0.09	-	99.4	0.4	0.03	0.08	-	-	0.06	-	-	-
Corundum	0.04	-	5.88	93.5	0.03	0.12	-	0.39	0.04	-	-	-
Chamotte	0.02	-	52.1	43.6	1.24	0.17	0.12	0.05	0.94	1.65	0.06	0.05
Cordierite	0.05	-	46.8	34.3	2.94	1.16	11.7	-	1.2	1.5	0.1	0.03

^a^ LOI = Loss on ignition.

**Table 2:** Physical properties of raw materials.

Material	Specific Gravity	Bulk Density	Particle Size		Specific Surface Area (BET)
	(kg/m^3^)	(kg/m^3^)	d50 (µm)	d90 (µm)	(m^2^/g)
Mefisto L_05_	2579	540	5.52	16.63	12.2
Quartz sand	2627	1564	-	-	-
Corundum	3892	2233	-	-	-
Chamotte	2541	1494	-	-	-
Cordierite	2487	1298	-	-	-
